# Ecology and phylogeny of birds foraging at outdoor restaurants in Sweden

**DOI:** 10.3897/BDJ.3.e6360

**Published:** 2015-09-24

**Authors:** Paul D. Haemig, Sara Sjöstedt de Luna, Henrick Blank, Henrik Lundqvist

**Affiliations:** ‡ Naturavdelningen, Länsstyrelsen i Jönköpings Län (English Translation: Nature Division, Governing Board of Jönköping Province), Jönköping, Sweden; § Institutionen för Matematik och Matematisk Statistik, Umeå Universitet (English Translation: Department of Mathematics and Mathematical Statistics, Umeå University), Umeå, Sweden

**Keywords:** Birds, cafés, conservation, ecology, ecophylogenetics, feeding associations, foraging associations, landscapes, restaurants, rural environments, urban environments

## Abstract

**Background:**

Birds frequently visit the outdoor serving areas of restaurants to feed on scraps of food and leftovers. Although this feeding association between humans and birds is widespread and could have significant effects, both positive and negative, for all taxa involved, the authors know of no published studies that have investigated restaurant bird communities. To lay the foundation for future research, the authors conducted a basic study of birds at 80 outdoor restaurants in Sweden, identifying which species and taxonomic clades of birds visited the restaurants and comparing restaurant birds in urban and rural environments.

**New information:**

Thirteen species of birds visited the outdoor restaurants. Eight of these species were predominant, i.e. accounting for 51% or more of bird presence (sum of minutes of all individual birds) at one or more restaurants. Every restaurant studied had a predominant species, but species often differed from each other in frequency of predominance in different landscapes. No endangered species were seen visiting restaurants. However, three farmland bird species (House Sparrow *Passer domesticus*, White Wagtail *Motacilla alba*, Eurasian Tree Sparrow *Passer montanus*), whose numbers are reported to be declining in the countryside, were predominant at the majority of restaurants in rural areas, suggesting that rural restaurants might be able to contribute to the conservation of these species. The thirteen species of restaurant-visiting birds belonged to five monophyletic clades. Ninety percent of all restaurants had, as their predominant species, birds from either Clade A (Passeridae, Motacillidae, Fringillidae) or Clade C (Corvidae). Statistical testing revealed that Clade A and Clade C were distributed differently in environments along the urban-rural gradient. At all spatial scales measured, birds of Clade C were predominant at the majority of restaurants in urban areas, while birds of Clade A were the predominant clade at the majority of restaurants in rural areas. The authors use this evidence, and observations of birds foraging in association with other primates, to hypothesize that the outdoor serving areas of modern restaurants may be helping to preserve and nurture ancient human-bird symbioses that have been part of human ecology since antiquity.

## Introduction

The sciences of ecology and ornithology seem to have completely overlooked outdoor restaurants and their avifauna. These forgotten microhabitats, scattered across the urban-rural gradient, host some of the most familiar birds known. Yet, the very “ordinariness” of their avifauna leads many people to take them for granted and wrongly assume that nothing new or important can be learned from studying them.

A good way to begin thinking about restaurants and their birds is to understand that they are part of a common ecological phenomena called “feeding associations” or “foraging associations”, where one species of animal feeds in association with another animal species to increase food intake and/or reduce mortality from predation (Barnett and Shaw 2014). In Africa, for example, Crested Guineafowls *Guttera pucherani* accompany groups of foraging monkeys and feed on fruits and fruit remains dropped or knocked to the ground by the monkeys (Hill 1974, Seavy et al. 2001).

The diversity of animal taxa that birds forage with is enormous, and these associations occur not only on land but also in freshwater and marine habitats, and from the tropics to the polar regions (Suppl. material 1). Included in the list of taxonomic orders that birds forage with are other bird species, ants, platypuses, aardvarks, armadillos, carnivores, ungulates, rodents, moles, fishes, turtles, crocodiles, cetaceans and pinnipeds (Suppl. material 1).

Among man’s closest relatives, the non-human primates, birds feed in association with at least sixteen genera of eight families (Table 1). For example, in the deep forests and remote marshes of the Congo region, Africa, at least five species of birds associate with the Western Lowland Gorilla *Gorilla gorilla gorilla*, feeding on insects and amphibians flushed when these great apes forage and move through vegetation (Ruggiero and Eves 1998).​

Considering these facts, it is not at all surprising then that birds seeking food also associate with humans. On busy city streets or along peaceful country roads, one regularly sees birds at open air restaurants, scavenging for food scraps and leftovers on tables, chairs and the ground below, often in close proximity to people. While some diners find these birds amusing and feed them, others resent the intrusion of uninvited feathered guests. Still other diners, however, are so busy socializing that they don’t even notice the birds.

Although human-bird feeding associations at outdoor restaurants are widespread, we found no published studies on this topic when we searched the scientific literature. Because these associations could conceivably influence humans and birds in many ways, both negatively and positively (e.g. public health), we think it important to study restaurants and their avifauna.

Accordingly, in 2013 and 2014, we initiated research on birds visiting restaurants. Our studies investigated the following basic questions: (1) Which bird species visit restaurants and which are most frequently seen there? (2) Which phylogenetic clades do restaurant birds belong to and which of these are most successful? (3) Are the bird species and clades that predominate at restaurants in the city the same as those that predominate at restaurants in the countryside? And finally, (4) do any endangered or declining bird species forage at outdoor restaurants, and are there any conceivable roles for restaurants in nature conservation?

In this paper, we use the terms “restaurant”, “outdoor restaurant” and “open air restaurant” as synonyms to mean specifically the outdoor serving area of a genuine restaurant or café, including its chairs, table and ground (usually a wooden deck, cement patio or grassy lawn). According to the definition we use here, a fence or wall surrounding a restaurant is not part of that restaurant.

## Methods

### Study sites

We studied birds at 80 outdoor restaurants in the following eight landscape provinces of Sweden: Bohuslän, Halland, Närke, Skåne, Småland, Värmland, Västergötland, Östergötland. The restaurants used as study sites were located in or near the following cities, towns, villages and settlements:

*Bohuslän*: Fiskebäckskil, Herrestad; *Halland*: Falkenberg, Gödestad, Halmstad, Tofta, Ullared, Varberg; *Närke*: Åmmeberg; *Skåne*: Mörarp; *Småland*: Alvesta, Aneby, Brunstorp, Eksjö, Gamlarp, Gränna, Hestra, Hestraviken, Hok, Huskvarna, Jönköping, Nässjö, Norra Glassbo, Norraby, Rönnhult, Sävsjö, Skedhult, Taberg, Tornaryd, Torsvik, Tranås, Vetlanda, Viredaholm, Värnamo; *Värmland*: Kristinehamn; *Västergötland*: Alingsås, Björkelund, Borås, Falköping, Göteborg, Hjo, Hulabäck, Karlsborg, Knistad, Lidköping vid Vänern, Lundsbrunn, Mariestad, Mölndal, Onsjö, Skara, Skövde, Stenkällegården, Tidaholm, Trollabo, Trollhättan, Ulricehamn, Viared, Vänersborg, Ålleberg; *Östergötland*: Klinga, Linköping, Motala, Mjölby, Norrköping; Stocklycke (Omberg), Stora Lund, Strå, Vadstena, Vida Vättern, Viringe, Väderstad, Ödeshög.

All restaurants studied were located at least one kilometer apart. Where more than one restaurant was available in an area, the restaurant chosen for sampling was selected by random lottery. Sampling was conducted when the restaurants were open.

### Bird presence

To measure and quantify the presence of each bird species, the outdoor serving area of each restaurant (tables, chairs, ground) was scanned every minute for one hour, and the number of individuals of each bird species seen in the restaurant during each minute was recorded. One “bird minute” was assigned for each minute that an individual bird was present on the tables, chairs and ground of the outdoor serving area. Only one of us (Haemig) gathered this data, and he sat at a table on the edge of each outdoor restaurant. Scan sampling was halted if a diner intentionally gave food to a bird, and restarted only after that diner left.

The length of the scan-sampling period was chosen in the following way: At seven restaurants, scan-sampling was conducted for several days and the data analysed. In each case, the identity of predominant bird species could be discerned within twenty minutes from the time sampling started. It was therefore deemed wiser (for a first study of restaurant birds) to increase the number of replicates (restaurants surveyed) than to do long-term sampling at a few restaurants. Nevertheless, just to be safe, the scan-sampling period for each restaurant was set at one hour per restaurant.

Later, when data collection was finished, the number of bird minutes at each restaurant was summed and the density calculated for each species as Bird Minutes/Hour/100 M^2^. The following example illustrates the method: Imagine that Bird Species A visited an outdoor restaurant of 100 M^2^ area during 15 minutes of an 8 hour sampling period. The counts of individuals of Species A seen in the restaurant during each of these 15 minutes was: 2, 3, 3, 3, 3, 3, 2, 2, 3, 3, 8, 6, 9, 9, 5. The sum of these minute counts is 64. Dividing by 8 (hours), Species A’s presence is calculated as 8 bird minutes per hour per 100 M^2^.

### Bird species and monophyletic clades

A list of all bird species seen visiting the restaurants was compiled and the phylogenetic clade of each bird species determined using Fjeldså (2013), a chapter in the *Handbook of the Birds of the World* that reviews and summarizes recent discoveries in the field of avian systematics, and proposes a plausible phylogeny (pp. 94-95) for all the world’s bird taxa based on the best available evidence at this time.

### Predominant species and predominant clades

At all restaurants, one bird species accounted for 51% or more of the total bird presence. We shall refer to this bird as the “predominant species” and the corresponding monophyletic clade with 51% or more of bird presence at the restaurant as the “predominant clade”. After the predominant species and clades were determined for each restaurant, the number of restaurants for each predominant species and clade were summed for urban, mixed and rural environments at different spatial scales (see below).

### Predominance in urban, mixed, and rural areas

At various spatial scales (see below), we classified the tract of land surrounding each restaurant as urban, rural or mixed. To do this, we used the ArcGIS^TM^ geographical information system to measure the area (m^2^) of various urban habitats surrounding each restaurant. We then divided this area by the total area of the tract being studied to obtain the percentage of urban habitats in each tract. If the amount of urban habitats in a tract was 0-33%, it was classified as “rural”. If the amount was 67-100%, it was “urban”. If it was 34-66%, it was classified as “mixed”.

For this classification, the following habitats from *Svenska Märktäckedata* (Naturvårdsverket 2014) were considered to be urban: crowded city neighborhoods covered more than 80% by buildings with hard, artificial outer surfaces; neighborhoods with more than 200 inhabitants (people) and small areas of gardens and green areas; neighborhoods with more than 200 inhabitants and large areas of gardens and green areas; settlements with less than 200 inhabitants but with 30-80% of buildings with hard outer surfaces; buildings on plots of non-agricultural land with open character; industrial areas, commercial districts, military garrisons; streets, highways and railroads; port and harbour facilities, airports and airfields; sand and gravel pits; other mining sites; waste disposal facilities; building sites, residential areas; urban green areas; sports facilities, rifle ranges, motor, dog and horseracing tracks.

### Spatial scales

The size of the habitat tracts surrounding each restaurant (see previous paragraph) were expanded in order to describe the environment at three different scales. From the center of each restaurant, circles were made with radii of 200 m, 500 m and 1000 m. The area of each tract was then determined by calculating the area of each circle.

## Results

### Bird species and phylogenetic clades

A total of 13 species of birds were observed visiting the outdoor serving areas of restaurants and foraging on food remains left by diners (Table 2, Suppl. material 2). The highest number of bird species recorded at a single restaurant was eight.

The highest density measured for a single species at a single restaurant was 485.29 bird minutes/hour/100M^2^ recorded for the House Sparrow (Suppl. material 2). Five other restaurants also had densities of House Sparrows that were higher than any other bird species in the entire study (Suppl. material 2).

Based on the phylogeny of Fjeldså 2013), the thirteen species of birds observed foraging at restaurants can be grouped into the following five monophyletic clades:

Clade A (Old World Sparrows *Passer*, Wagtails *Motacilla*, Finches *Fringilla*)

Clade B (Tits *Parus*)

Clade C (Jackdaws *Coloeus*, Crows and Rooks *Corvus*, Magpies *Pica*,

Clade D (Pigeons *Columba*)

Clade L (Gulls *Larus*)

The first three clades are songbirds (Order Passeriformes). Clade A includes species from three closely-related families (Passeridae, Motacillidae, Fringillidae). Although wagtails *Motacilla* seem very different from Old World Sparrows *Passer* in appearance and behavior, recent molecular studies show that they are closely related (Voelker and Edwards 1998). The birds of Clade B belong to the family Paridae (tits and chickadees). Clade C includes species from three closely-related Old World corvid genera (Ericson et al. 2005). The last two clades (D and L) are non-passerine birds of the familes Columbidae and Laridae. Table 2 lists the clade for each of the thirteen species of restaurant-visiting birds.

### Predominant bird species and clades

Eight of the thirteen restaurant bird species were found to be predominant species (Table 2). That is, each of these eight species accounted for 51% or more of bird presence in the outdoor serving area of at least one restaurant. Most predominant species were passerines (Table 2).

Four of the five clades were predominant at one or more restaurants (Table 3). Two clades (A and C) were predominant at the overwhelming majority (90%) of restaurants (Table 3).

Only one clade (L) was never found to be predominant (Tables 2, 3). However, our methodology may have led to underestimation of this clade. At restaurants in this study, the birds of Clade L (gulls) behaved like raptors. They frequently flew over and circled over many restaurants, or perched on nearby roofs or lamp posts, all the while observing closely the food situation in the restaurant. When a food item to their liking was left unattended, the gulls would swoop down and land for a short time, quickly seize the food and then either rapidly eat it in the restaurant or fly away with it. Because we measured bird presence only when a bird was on a table, chair or the ground of the restaurant, a typical gull visit to a restaurant was recorded as being one minute. However, because the gulls were observing the dining area for long periods of time while they were outside the restaurant, a different definition of presence might have increased the measure of bird presence for gulls.

### Distribution of predominant species at restaurants in different environments

Statistical testing at all scales confirmed that the Eurasian Jackdaw *Coloeus monedula* and White Wagtail *Motacilla alba* were distributed differently along the urban-rural gradient (Table 4). In urban areas, at all spatial scales, the Eurasian Jackdaw was predominant at over half the restaurants (Table 2). In contrast, the White Wagtail was the species that most often predominated at restaurants in rural areas and was predominant mainly there (Table 2). Similar patterns were found when comparing the Eurasian Jackdaw with either the Great Tit *Parus major* or the Eurasian Tree Sparrow *Passer montanus*, the latter two species being predominant mainly in rural areas (Tables 2, 4).

The House Sparrow predominated at many restaurants in both rural and urban areas (Table 2). Statistical testing at all scales revealed that its distribution on the urban-rural gradient was significantly different from that of the Great Tit, White Wagtail, and Eurasian Tree Sparrow, but not the Eurasian Jackdaw (Table 4).

### Distribution of predominant clades at restaurants in different environments

At all scales measured, Clade C (Corvidae) was predominant at 56.3 to 58.3% of restaurants in urban areas, while Clade A (Passeridae, Motacillidae, Fringillidae) was predominant at 65.9 to 73.0% of restaurants in rural areas (Table 3). Statistical testing confirmed that these two clades were distributed differently in urban, mixed and rural areas (Table 4).

Clade B (Paridae) showed a pattern similar to Clade A (Table 3). It predominated mainly at restaurants in rural areas and statistical testing confirmed that its distribution differed significantly from Clade C (Table 4).

### Endangered and declining bird species

No endangered bird species were seen visiting the restaurants (Table 2). However, three species of declining farmland birds were predominant at the majority of restaurants in rural areas (Table 2).

The first of these declining farmland birds, the House Sparrow, decreased -73% in Swedish farmlands between 1976 and 2001 (Wretenberg et al. 2006). In the present study, this species was predominant at 18.2% to 24.3% of restaurants in rural areas (Table 2).

The second species of declining farmland bird, the Eurasian Tree Sparrow, decreased -25% in Swedish farmlands between 1976 and 2001 (Wretenberg et al. 2006). In the present study, it was predominant at 9.8% to 12.2% of restaurants in rural areas (Table 2).

The third species, the White Wagtail, declined -22%, in Swedish farmlands between 1976 and 2001 (Wretenberg et al. 2006). In the present study, the White Wagtail was predominant at 29.3% to 36.4% of restaurants in rural areas (Table 2).

At the 200 meter scale, two thirds (66.8%) of rural restaurants had one of these three declining farmland birds as its predominant species (Table 2). Similarly, at the 500 and 1000 meter scales, 67.5% and 61.1% of the rural restaurants had one of these birds as its predominant species (Table 2).

## Discussion

Only thirteen bird species were seen visiting the restaurants, and only eight bird species were predominant there. This low diversity of bird species is remarkable when one considers the large number of birds living in Sweden.

The thirteen species of restaurant-visiting birds come from only five monophyletic clades, and this fact is also remarkable. The low number of clades suggests that there is a phylogenetic component to the propensity of some bird species to enter into feeding associations with humans. It also suggests that it may not be easy for most wild birds to develop adaptations for participating in close, intimate, frequent, dining relationships with *Homo sapiens*, the most dangerous and unpredictable of all species.

If, as our results indicate, evolution has played an important role in the development of the feeding associations we studied between birds and humans, the question naturally arises as to how long each clade’s feeding association with humans has existed. At least one clade’s association seems to have begun recently. The Feral Pigeon (Clade D) is a bird whose presence at restaurants can be explained simply by the fact that it once was domesticated and is now feral. However, the fact that this species was once domesticated might, on the contrary, mean that it already had a long association with humans before domestication and at that time evolved traits and behaviors that pre-adapted it to domestication.

The enormous number of feeding associations of birds with other taxa (Suppl. material 1), including primates (Table 1), suggests the possibility that, in the past, birds foraged with some of our human and pre-human ancestors. This possibility, combined with the above indications that evolution has played a role in bird-human feeding associations, suggests that at least some of the associations of birds with humans at outdoor restaurants today might be very ancient symbioses, perhaps even predating the emergence of modern humans. They might also predate the emergence of some of the bird species that forage at restaurants today, and of course be much older than the restaurants themselves.

In this regard, we would like to draw the attention of the reader to Clade A, which we consider to be the most successful of the clades that we studied because it was predominant at so many restaurants in all three environments (urban, mixed, rural), and at all scales measured (Table 3). This clade has evolved into at least three families (Passeridae, Motacillidae, Fringilidae) and is also the most successful clade in the oldest environment occupied by humans: the rural countryside. These facts suggest that the association of Clade A with humans might be very ancient, and we should even consider the possibility that earlier bird species of this clade fed in association with one or more of our fossil hominoid ancestors or, alternatively, with other prehistoric animals and then later switched to humans or earlier hominoids.

The association with Clade C (Corvidae) may also be much older than commonly assumed. It has diverged into at least three genera and, while *Corvus* and *Pica* are more shy and may simply be generalized scavengers, *Coloeus* behaves very much like an “old friend” of human diners. At many restaurants, this bird acts familiar to the verge of impudence and its sometimes amusing, comical behaviour might be an adaptation for manipulating humans to feed it, or at least to accept its close presence. Nevertheless, any historical and evolutionary reconstruction of human-bird feeding associations at restaurants needs to explain why not all bird species in the taxonomic families of the five clades were seen visiting restaurants (e.g. *Anthus* spp.).

Now that we have identified the key players (bird species and clades) that forage at restaurants in various landscapes of southern Sweden, other important questions can now be investigated. For example, we could ask to what extent restaurants and the birds foraging at them impact, ecologically and evolutionarily, individual organisms, species, populations, communities and ecosystems where they live.

Restaurants regularly supply food subsidies to wild birds so, using the definition of Oro et al. 2013, restaurants could be classified and understood as predictable anthropogenic food sources (PAFS). Other PAFS that have been studied alter bird behaviour and body condition, individual life-history traits such as survival, reproduction and dispersal, and population traits such as density and size (Robb et al. 2008, Jones 2011, Oro et al. 2013, Amrhein 2014, Galbraith et al. 2014). These changes sometimes “result in cascading effects across non-adjacent population levels, pervading whole ecosystems with potential impacts on stability, flexibility and persistence” (Oro et al. 2013).

While the present study did not quantitatively investigate such impacts, there is good reason to believe that they may be significant. For example, one result of bird foraging at restaurants is efficient diurnal cleaning of the outdoor serving area and, consequently, a great reduction in the amount of food remains available to nocturnal scavengers such as rodents. The senior author, who conducted the fieldwork of the present study, often saw birds at numerous restaurants clean up every scrap and crumb of food in sight with an efficiency that was truly amazing.

By cleaning restaurants in this way and thereby likely reducing the density of rodents where humans eat, birds provide not only a free janitorial service but also could be performing an important public health service. Rodents carry serious zoonotic infections far more frequently than do birds and, because rodents are also disease reservoirs, sustaining and amplifying some of the most feared microorganisms known to humans, they are generally a far greater menace to humans than restaurant birds, which in comparison are usually only accidental and/or less-frequent hosts and reservoirs of these microbes.

### Nature conservation and outdoor restaurants

Although no endangered species were seen foraging at outdoor restaurants in this study, restaurants located in rural areas supported three species of declining farmland birds to such an extent that these three species were predominant at the majority of restaurants in the countryside. One wonders, therefore, if there might be some role for rural restaurants to play in conservation programs to preserve these species in increasingly hostile agrarian landscapes.

In addition, if any of the human-bird associations that occur at restaurants are ancient (as discussed above), then there may be another important conservation role for the outdoor restaurant besides preservation of declining farmland bird species. In many countries today humans eat mainly indoors. Yet, in the past, humans and their fossil hominoid ancestors ate mainly outdoors. The modern restaurant with its outdoor serving area might therefore be helping to preserve and nurture ancient symbioses between birds and humans that have been part of human ecology since antiquity.

## Supplementary Material

Supplementary material 1 Appendix - Known feeding associations of birds with other animal species Data type: Symbioses
Brief description: Some of the animals with which birds form feeding associations. Although incomplete, this list shows that foraging birds associate with a diversity of animal taxa and that such associations occur in terrestrial, freshwater and marine environments, and from the tropics to the polar regions. For feeding associations of birds with non-human primates see Table 1 (this paper).
File: oo_50080.docx Paul D. Haemig, Sara Sjöstedt de Luna, Henrick Blank, Henrik Lundqvist

Supplementary material 2 Densities of birds at outdoor restaurants in Sweden Data type: Occurences
Brief description: The density of birds at each restaurant surveyed, with percentage of urbanization of the environment surrounding each restaurant at the 200 meter, 500 meter and 1000 meter radius scales.
File: oo_50081.xlsx Paul D. Haemig, Sara Sjöstedt de Luna, Henrick Blank, Henrik Lundqvist

## Figures and Tables

**Table 1.** Examples of non-human primates with which birds form feeding associations. The bird species consorting with each primate taxa are listed in the reference(s) beside each primate genus. For theoretical aspects of these associations see Barnett and Shaw 2014, Heymann and Hsia 2015, Terborgh 1990.

**Order PRIMATES**	
**Family LEMURIDAE**	
Bamboo Lemurs *Hapalemur*	Eppley et al. 2014
**Family CALLITHRICHIDAE**	
Lion tamarins *Leontopithecus*	Hankerson et al. 2006, Kuniy et al. 2003
Tamarins *Saguinus*	Egler 1991, Heymann 1992, Siegel et al. 1989
Marmosets *Callithrix*	Ferrari 1990
**Family CEBIDAE**	
Squirrel monkeys *Saimiri*	Boinski and Scott 1988
Capuchin monkeys *Cebus*	Fontaine 1980, Rodrigues et al. 1994, Zhang and Wang 2000
**Family PITHECIIDAE**	
Uacaris *Cacajao*	Barnett and Shaw 2014
**Family ATELIDAE**	
Howlers *Alouatta*	Rodrigues et al. 1994
**Family CERCOPITHECIDAE**	
Macaques *Macaca*	Matsumura 2001, Stott 1947
Mangabeys *Lophocebus*	Seavy et al. 2001
Baboons *Papio*	King and Cowlishaw 2009
*Chlorocebus* monkeys	Hill 1974
*Cercopithecus* monkeys	Seavy et al. 2001
*Colobus* monkeys	Dean and MacDonald 1981, Ruggiero and Eves 1998
**Family HYLOBATIDAE**	
Gibbons *Hylobates*	Gaietti and McConkey 1998
**Family HOMINIDAE**	
Gorillas *Gorilla*	Ruggiero and Eves 1998

**Table 2.** Bird species found visiting outdoor restaurants in Sweden. The thirteen species detected belonged to five monophyletic clades (A, B, C, D, L). Eight species were predominant species, i.e. accounted for 51% or more of the total bird presence at one or more of the eighty restaurants studied during the censuses. The final nine columns of the table show the number of restaurants where each of these eight species was predominant in various landscapes (urban, mixed, rural). The data are shown at three different scales of area (circles with radii of 200 Meters, 500 Meters and 1000 Meters from the center of the outdoor serving area of each restaurant). Below every number are the percentages of that number in each column.

**Bird Species**	**Monophyletic**	**200 Meter Radius**					**500 Meter Radius**			**1000 Meter Radius**			
**(*Latin Name*, Swedish Name)**	**Clade**	**Urban**		**Mixed**	**Rural**		**Urban**	**Mixed**	**Rural**	**Urban**		**Mixed**	**Rural**
House Sparrow (*Passer domesticus*, Gråsparv)	A	13 40.6%		6 40.0%	6 18.2%		12 41.4%	4 28.6%	9 24.3%	9 37.5%		7 46.7%	9 22.0%
Eurasian Tree Sparrow (*Passer montanus*, Pilfink)	A	-		-	4 12.2%		-	-	4 10.8%	-		-	4 9.8%
White Wagtail (*Motacilla alba*, Sädesärla)	A	-		1 6.67%	12 36.4%		-	1 7.1%	12 32.4%	-		1 6.7%	12 29.3%
Common Chaffinch (*Fringilla coelebs*, Bofink)	A	-		-	2 6.1%		-	-	2 5.4%	-		-	2 4.9%
Great Tit (*Parus major*, Talgoxe)	B	-		1 6.67%	6 18.2%		-	1 7.1%	6 16.2%	-		-	7 17.1%
Eurasian Jackdaw (*Coloeus monedula*, Kaja)	C	17 53.1%		6 40.0%	2 6.1%		15 51.7%	7 50.0%	3 8.1%	13 54.2%		6 40.0%	6 14.6%
Common Magpie (*Pica pica*, Skata)	C	1 3.1%		1 6.67%	1 3.0%		1 3.4%	1 7.1%	1 2.7%	1 4.2%		1 6.7%	1 2.4%
Hooded Crow (*Corvus cornix*, Kråka)	C	-		-	-		-	-	-	-		-	-
Rook (*Corvus frugilegus*, Råka)	C	-		-	-		-	-	-	-		-	-
Rock Dove (*Columba livia*, Stadsduva)	D	1 3.1%		-	-		1 3.4%	-	-	1 4.2%		-	-
Mew Gull (*Larus canus*, Fiskmås)	L	-		-	-		-	-	-	-		-	-
Black-headed Gull (*Larus ridibundus*, Skrattmås)	L	-		-	-		-	-	-	-		-	-
Herring Gull (*Larus argentatus*, Gråtrut)	L	-		-	-		-	-	-	-		-	-

**Table 3.** Number of restaurants where each monophyletic clade was predominant in different landscapes (urban, mixed, rural) along the urban-rural gradient at various scales of area. Below every number are the percentages of that number in each column. Note: this is the same data as Table 2, but here the data have been grouped by clade rather than species.

**Monophyletic**	**200 Meter Radius**			**500 Meter Radius**			**1000 Meter Radius**		
**Clade**	**Urban**	**Mixed**	**Rural**	**Urban**	**Mixed**	**Rural**	**Urban**	**Mixed**	**Rural**
A	13 40.6%	7 46.6%	24 72.7%	12 41.4%	5 35.7%	27 73.0%	9 37.5%	8 53.3%	27 65.9%
B	-	1 6.7%	6 18.2%	-	1 7.1%	6 16.2%	-	-	7 17.1%
C	18 56.3%	7 46.7%	3 9.1%	16 55.2%	8 57.1%	4 10.8%	14 58.3%	7 46.7%	7 17.1%
D	1 3.1%	-	-	1 3.4%	-	-	1 4.2%	-	-
L	-	-	-	-	-	-	-	-	-

**Table 4.** Statistical comparisons of distributions of bird species found visiting outdoor restaurants in Sweden. All data tested are from Tables 2 and 3. Comparisons of species and clades not shown here either had too small sample sizes and/or their results were not statistically significant.

**Taxa Tested**	**Table**	**Statistical Test**	**200 Meter Radius**	**500 Meter Radius**	**1000 Meter Radius**
Eurasian Jackdaw *versus* White Wagtail	2	Fisher’s Exact Test (Urban + Mixed) *versus* Rural	*p* < 0.0001	*p* < 0.0001	*p* < 0.0001
Eurasian Jackdaw *versus* House Sparrow	2	Fisher’s Exact Test (Two-tailed) (Urban + Mixed) *versus* Rural	Not Significant (*p* = 0.247)	Not Significant (*p* = 0.096)	Not Significant (*p* = 0.538)
Eurasian Jackdaw *versus* Eurasian Tree Sparrow	2	Fisher’s Exact Test (Two-tailed) (Urban + Mixed) *versus* Rural	*p* < 0.001	*p* < 0.001	*p* < 0.01
Eurasian Jackdaw *versus* Great Tit	2	Fisher’s Exact Test (Two-tailed) (Urban + Mixed) *versus* Rural	*p* < 0.001	*p* < 0.001	*p* < 0.001
House Sparrow *versus* Great Tit	2	Fisher’s Exact Test (Two-tailed) (Urban + Mixed) *versus* Rural	*p* < 0.01	*p* < 0.05	*p* < 0.01
House Sparrow *versus* White Wagtail	2	Fisher’s Exact Test (Two-tailed) (Urban + Mixed) *versus* Rural	*p* < 0.0001	*p* < 0.01	*p* < 0.01
House Sparrow *versus* Eurasian Tree Sparrow	2	Fisher’s Exact Test (Two-tailed) (Urban + Mixed) *versus* Rural	*p* < 0.01	*p* < 0.05	*p* < 0.05
Clade A *versus* Clade C	3	Chi-Square (*x*^2^) Test for Independence Urban *versus* Mixed *versus* Rural Degrees of Freedom = 2	*x*^2^ = 14.29 *p* < 0.001	*x*^2^ = 15.54 *p* < 0.001	*x*^2^ = 9.849 *p* < 0.01
Clade B *versus* Clade C	3	Fisher’s Exact Test (Urban + Mixed) *versus* Rural	*p* < 0.001	*p* < 0.001	*p* < 0.001
