# Supplementary material for: Ecology and phylogeny of birds foraging at outdoor restaurants in Sweden
Source: Biodivers Data J. 2015 Sep 24;(3):e6360. doi: 10.3897/BDJ.3.e6360 (PMC4609751; doi:10.3897/BDJ.3.e6360)
Supplement: Supplementary material 1 — Appendix - Known feeding associations of birds with other animal species [file biodiversity_data_journal-3-e6360-s001.docx]

**Appendix** Some of the animals with which birds form feeding associations. Although incomplete, this list shows that foraging birds associate with a diversity of animal taxa and that such associations occur in terrestrial, freshwater and marine environments, and from the tropics to the polar regions. For feeding associations of birds with non-human primates see Table 1 (this paper).

| **Class** | **Order** | **Terrestrial** | **Freshwater** | **Marine** |
| --- | --- | --- | --- | --- |
| INSECTA  (Insects) | Hymenoptera | Army Ants *Eciton*  (Coates Estrada & Estrada 1989)  (Haemig 1989, Wrege *et al.* 2005)  Safari Ants *Anomma*  (Willis 1985, 1986) |  |  |
| ACTINOPTERYGII  (Ray-finned Fishes) | Perciformes |  | Freshwater Fish  (Ubaid 2011) | Skipjack Tuna *Katsuwonus*  (Hebshi *et al.* 2008) |
| TESTUDINES  (Turtles) | Chelonii |  | Freshwater Turtles  (Gosper 2008) | Sea Turtles *Lepidochelys*  (Pitman 1993) |
| REPTILIA  (Reptiles) | Crocodilia | Crocodiles *Crocodilus*  (Attwell 1966) |  |  |
| AVES  (Birds) | Struthioniformes | Ostriches *Struthio*  (Kamler *et al.* 2008) |  |  |
|  | Anseriformes |  | Swans *Cygnus*  (Gyimesi *et al.* 2012) |  |
|  | Galliformes | Wild Turkeys *Meleagris*  (Baker 1980) |  |  |
|  | Ciconiiformes |  | Ibises *Eudocimus*  (Kushlan 1978) |  |
|  | Charadriiformes | Auklets *Cerorhinca*  (Grover & Olla 1983) |  |  |
|  | Piciformes | Flickers *Colaptes*  (Short 1969) |  |  |
|  | Passeriformes | Migrant songbirds  (Renfrew 2007) |  |  |
| MAMMALIA  (Mammals) | Monotremata |  | Platypus *Ornithorhynchus*  (Troughton & Wray 1994) |  |
|  | Tubulidentata | Aardvarks *Orycteropus*  (Taylor & Skinner 2001) |  |  |
|  | Proboscidea | Elephants *Loxodonta*  (Rice 1963) |  |  |
|  | Sirenia |  | Manatees *Trichechus*  (Scott & Powell 1982) |  |
|  | Cingulata | Armadillos *Dasypus, Euphractus*  (Komar & Hanks 2002)  (Di Giacomo & Di Giacomo 2006) |  |  |
|  | Primates | See Table 1 |  |  |
|  | Rodentia | Squirrels *Urosciurus*  (Della-Flora *et al.* 2013)  Capybaras *Hydrochoerus*  (Tomazzoni 2005) |  |  |
|  | Soricomorpha | Moles *Talpa*  (McCanch & McCanch 1982, Sharrock 1982) |  |  |
|  | Carnivora | Aardwolves *Proteles*  (Anderson 1992)  Mongooses *Helogale*  (Rasa 1983)  Bat-eared Foxes *Otocyon*  (Stenkewitz & Kamler 2008)  Maned Wolves *Chrysocyon*  (Silveira 1997)  Coatis *Nasua*  (Beisiegel 2007) | Otters *Lontra, Lutrogale*  (D’Angelo & Sazima 2014)  (Kruuk *et al.* 1993) | Fur Seals *Arctocephalus*  (Thiebot & Weimerskirch 2012) |
|  | Perissodactyla | Zebras *Equus*  (Dean & MacDonald 1981)  Rhinoceroses *Ceratotherium, Diceros*  (Dean & MacDonald 1981) |  |  |
|  | Artiodactyla | Hippopotamuses *Hippopotamus*  (Dean & MacDonald 1981)  Warthogs *Phacochoerus*  (Dean & MacDonald 1981)  Deer *Rangifer, Odocoileus,*  (Pedersen *et al.* 2006 Herring & Herring 2007)  Cattle *Bos*  (Källander 1993)  Giraffes *Giraffa*  (Dean & MacDonald 1981) |  |  |
|  | Cetacea | Bottlenose Dolphins *Tursiops*  (Fox & Young 2012) |  | Gray Whale *Eschrichtius*  (Harrison 1979)  Minke Whales *Balaenoptera*  (Thiebot & Weimerskirch 2013)  Sperm Whales *Physeter*  (Thiebot & Weimerskirch 2013)  Killer Whales *Orcinus orca*  (Ridoux 1987)  Ocean Dolphins *Stenella, Lagenorhynchus*  (Thiebot and Weimerskirch 2013)  Bottlenose Whales *Hyperoodon*  (Thiebot & Weimerskirch 2013) |

**REFERENCES**

Anderson, M.D. (1992). Anteating chats feeding in association with aardwolves. *Ostrich*, 63, 186.

Attwell, R.I.G. (1966). Possible bird-crocodile commensalism in Zambia. *Ostrich*, 37, 54-55.

Baker, B.W. (1980). Commensal foraging of Scissor-tailed Flycatchers with Rio Grande Turkeys. *Wilson Bulletin*, 92: 248.

Beisiegel, B. de Mello (2007). Foraging association between Coatis (*Nasua nasua*) and birds of the Atlantic Forest, Brazil. *Biotropica*, 39, 283-285.

Coates Estrada, R. & Estrada, A. (1989). Avian attendance and foraging at army-ant swarms in the tropical rainforest of Los Tuxtlas, Veracruz, Mexico. *Journal of Tropical* Ecology, 5, 281-292.

Dean, W.R.J. & MacDonald, I.A.W. (1981). A review of African birds feeding in association with mammals. *Ostrich*, 52, 135-155.

Della-Flora, F., Melo, G.L., Sponchiado, J. & Cáceres, N.C. (2013). Association of the southern Amazon Red Squirrel *Urosciurus spadiceus* Olfers, 1818 with mixed species bird flocks. *Mammalia*, 77, 113-117.

Di Giacomo, A.S. & Di Giacomo, A.G. (2006). Observations of Strange-tailed Tyrants (*Alectrurus risora*) and other grassland birds following army ants and armadillos. *Journal of Field Ornithology*, 77, 266-268.

D’Angelo, G.B. & Sazima, I. (2014). Commensal association of piscivorous birds with foraging otters in southeastern Brazil, and a comparison with such a relationship of piscivorous birds with cormorants. *Journal of Natural History*, 48, 241-249.

Fontaine, R. (1980). Observations on the foraging association of Double-toothed Kites and White-faced Capuchin Monkeys. *Auk,* *97*, 94-98.

Fox, A.G. & Young, R.F. (2012). Foraging interactions between wading birds and strand-feeding bottlenose dolphins (*Tursiops truncatus*) in a coastal salt marsh. *Canadian Journal of Zoology*, 90, 744-752.

Gosper, C.R. (2008). A foraging association between an Australasian Grebe and a freshwater turtle. *Australian Field Ornithology*, 25, 46.

Grover, J.J. & Olla, B.L. (1983). The role of the Rhinoceros Auklet (*Cerorhinca monocerata*) in mixed-species feeding assemblages of seabirds in the Strait of Juan de Fuca, Washington. *Auk*, 100, 979-982.

Gyimesi, A., van Lith, B. & Nolet, B.A. (2012). Commensal foraging with Bewick’s Swans *Cygnus bewickii* doubles instantaneous intake rate of Common Pochards *Aythya ferina*. *Ardea*, 100, 55-62.

Haemig, P.D. (1989). Brown Jays as army ant followers. *Condor*, 91, 1008-1009.

Harrison, C.S. (1979). The association of marine birds and feeding gray whales. *Condor*, 81, 93-95.

Hebshi, A.J., Duffy, D.C. Hyrenbach K.D. (2008). Associations between seabirds and subsurface predators around Oahu, Hawaii. *Aquatic Biology*, 4, 89-98.

Herring, G. & Herring, H.K. (2007). Commensal feeding of Great Egrets with Black-tailed Deer. *Western Birds*, 38, 299-302.

Kamler, J.F., Suinyuy, T.N. & Goulding, W. (2008). Cattle Egret and Common Ostrich associations in South Africa. *Ostrich*, 79, 105-106.

Komar, O. & Hanks, C.K. (2002). Fan-tailed Warbler foraging with nine-banded armadillos. *Wilson Bulletin*, 114, 526-528.

Kruuk, H., Kanchanasaka, B., O’Sullivan, S. & Wanghongsa, S. (1993). Kingfishers *Halcycon capensis* and *Alcedo atthis* and Pond-heron *Ardeola bacchus* association with otters *Lutra perspicillata*. *Natural History Bulletin of the Siam Society*, 41, 67-68.

Kushlan, J.A. (1978). Commensalism in the Little Blue Heron. *Auk*, 95, 677-681.

Källander, H. (1993). Commensal feeding associations between Yellow Wagtails *Motacilla flava* and cattle. *Ibis*, 135, 97-100.

McCanch, N.V., & McCanch M. (1982). Feeding association between mole and juvenile song thrush. *British Birds*, 75, 90.

Pedersen, A.Ø., Lier, M., Routti, H., Christiansen, H.H. & Fuglei, E. (2006). Co-feeding between Svalbard Rock Ptarmigan (*Lagopus mutus hyperboreus*) and Svalbard Reindeer (*Rangifer tarandus platyrhynchus*). *Arctic*, 59, 61-64.

Pitman, R.L. (1993). Seabird associations with marine turtles in the eastern Pacific. *Colonial Waterbirds*, 16, 194-201.

Rasa, O.A.E. (1983). Dwarf Mongoose and hornbill mutualism in the Taru Desert, Kenya. *Behavioral Ecology and Sociobiology*, 12, 181-190.

Renfrew, R.B. (2007). An interspecific foraging association between Nearctic-Neotropical migrant passerines in Bolivia. *Wilson Journal of Ornithology*, 119, 124-126.

Rice, D.W. (1963). Birds associating with elephants and hippopotamuses. *Auk*, 80, 196-197.

Ridoux, V. (1987). Feeding association between seabirds and killer whales, *Orcinus orca,* around sub-Antarctic Crozet Islands. *Canadian Journal of Zoology*, 65, 2113-2115.

Ruggiero, R.G. & Eves, H.E. (1998). Bird-mammal associations in forest openings of northern Congo (Brazzavile). *African Journal of Ecology*, 36, 183-193.

Scott, M.D. & Powell, J.A. (1982). Commensal feeding of Little Blue Herons with Manatees. *Wilson Bulletin*, 94, 215-216.

Sharrock, J.T.R. (1982). Feeding association between mole and birds. *British Birds*, 75, 90.

Short, L.L. (1969). Foraging association of Green-barred Flickers and Campo Flickers in Argentina. *Wilson Bulletin*, 81, 468-470.

Silveira, L., Jácomo, A.T.A., Rodrigues, F.H.G. & Crawshaw. P.G. (1997). Hunting association between the Aplomado Falcon (*Falco femoralis*) and the Maned Wolf (*Chrysocyon brachyurus*) in Emas National Park, Central Brazil. *Condor*, 99, 201-202.

Stenkewitz, U. & Kamler, J.F. (2008). Birds feeding in association with bat-eared foxes on Benfontein Game Farm, South Africa. *Ostrich*, 79, 235-237.

Taylor, W.A. & Skinner, J.D. (2001). Associative feeding between Ant-eating Chats, *Myrmecocichla formicivora*, and Aardvarks, *Orycteropus afer*. *Ostrich*, 72, 199-218.

Thiebot, J-B. & Weimerskirch, H. (2013). Contrasted associations between seabirds and marine mammals across four biomes of the southern Indian Ocean. *Journal of Ornithology*, 154, 441-453.

Tomazzoni, A.C., Pedo, E. & Hartz, S.M. (2005). Feeding associations between capybaras *Hydrochoerus hydrochaeris* (Linneaus) (Mammalia, Hydrochaeridae) and birds in the Lami Biological Reserve, Porto Alegre, Rio Grande do Sul, Brazil. *Revista Brasileira de Zoologia*, 22, 713-716.

Troughton, G.J. & Wray, S. (1994). An apparent feeding association between Azure Kingfisher *Ceyx azurea* and the platypus *Ornithorhynchus anatinus*. *Sunbird*, 24, 45.

Ubaid, F.K. (2011). Greater Anis (*Crotophaga major*) commensal foraging with freshwater fish in the Pantanal Floodplain, Brazil. *Wilson Journal of Ornithology*, 123, 171-173.

Willis, E.O. (1985). East African Turdidae as safari ant followers. *Le Gerfaut*, 75:,140-153.

Willis, E.O. (1986). West African thrushes as safari ant followers. *Le Gerfaut*,76, 95-108.

Wrege, P.H., Wikelski, M., Mandel, J.T., Rassweiler, T. & Couzin, I.D. (2005). Antbirds parasitize foraging army ants. *Ecology*, 86, 555-559.
